# Retinal determination genes coordinate neuroepithelial specification and neurogenesis modes in the *Drosophila* optic lobe

**DOI:** 10.1242/dev.135004

**Published:** 2016-07-01

**Authors:** Holger Apitz, Iris Salecker

**Affiliations:** The Francis Crick Institute, Mill Hill Laboratory, London NW7 1AA, UK

**Keywords:** Visual system, Neuroepithelial specification, Neurogenesis, *Polycomblike*, Retinal determination genes, *Drosophila*

## Abstract

Differences in neuroepithelial patterning and neurogenesis modes contribute to area-specific diversifications of neural circuits. In the *Drosophila* visual system, two neuroepithelia, the outer (OPC) and inner (IPC) proliferation centers, generate neuron subtypes for four ganglia in several ways. Whereas neuroepithelial cells in the medial OPC directly convert into neuroblasts, in an IPC subdomain they generate migratory progenitors by epithelial-mesenchymal transition that mature into neuroblasts in a second proliferative zone. The molecular mechanisms that regulate the identity of these neuroepithelia, including their neurogenesis modes, remain poorly understood. Analysis of *Polycomblike* revealed that loss of Polycomb group-mediated repression of the Hox gene *Abdominal-B* (*Abd-B*) caused the transformation of OPC to IPC neuroepithelial identity. This suggests that the neuroepithelial default state is IPC-like, whereas OPC identity is derived. Ectopic Abd-B blocks expression of the highly conserved retinal determination gene network members Eyes absent (Eya), Sine oculis (So) and Homothorax (Hth). These factors are essential for OPC specification and neurogenesis control. Finally, *eya* and *so* are also sufficient to confer OPC-like identity, and, in parallel with *hth*, the OPC-specific neurogenesis mode on the IPC.

## Abstract (precis)

**Summary:** Polycomb-mediated repression of the *Abd-B* Hox gene controls expression of retinal determination genes and hence identity of the *Drosophila* optic lobe neuroepithelia.

## INTRODUCTION

Mature brains are built of interconnected neural circuits, development of which commonly begins with simple neuroectodermal or neuroepithelial (NE) sheets. In an initial proliferative phase, NE cells expand by symmetric cell divisions and become patterned into discrete territories. During a subsequent neurogenic phase, NE cells convert into neural stem cells (NSCs), which undergo asymmetric self-renewing divisions to produce diverse neuronal and glial subtypes (Paridaen and Huttner, 2014). Recent studies in vertebrates uncovered additional cortical neural stem and progenitor cell types with distinct morphologies, division modes and cellular behaviors that contribute to area- and species-specific differences in neurogenesis (Taverna et al., 2014). Similarly, in *Drosophila*, several neurogenesis modes have been identified in the embryonic and postembryonic brain and ventral nerve cord (VNC) (Yasugi and Nishimura, 2015). However, the molecular mechanisms that potentially could link region-specific patterning and neurogenesis modes remain poorly understood.

**Fig. 1.**
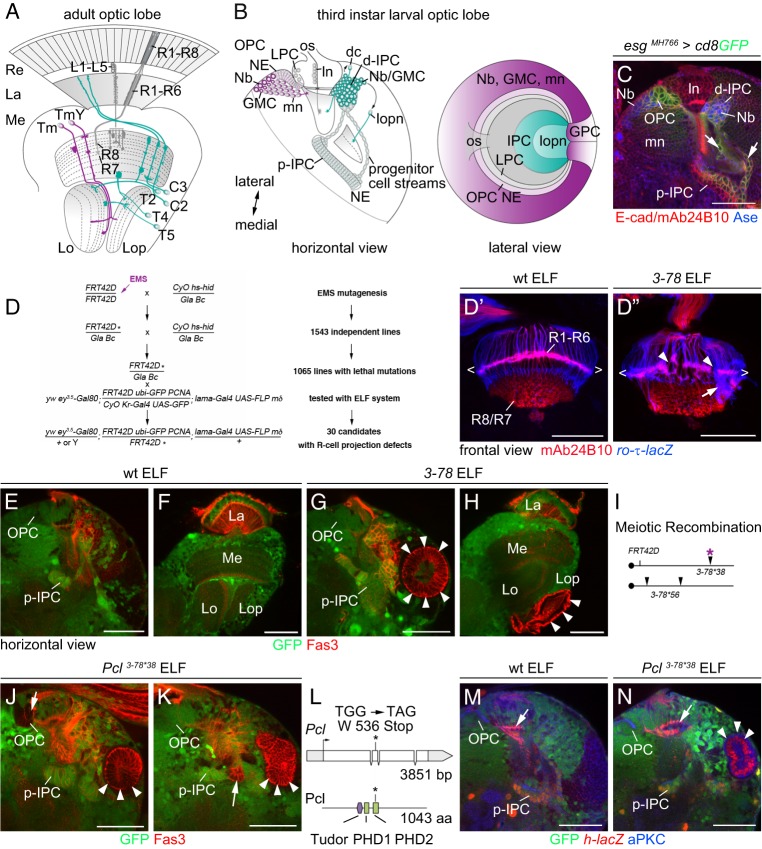
**Identification of *Pcl* in a screen for genes controlling NE patterning.** (A,B) Schematic of adult (A) and third instar larval (B) *Drosophila* optic lobes in horizontal and lateral views. The outer and inner proliferation centers (OPC, IPC) and progeny are shown in magenta and green, respectively. (C) In third instar larval optic lobes, *esg^MH766^-Gal4, UAS-cd8GFP* (green) label the OPC, IPC and their progeny, E-cad (red) cell membranes, and mAb24B10 (red) R1-R8 axons. Nbs and GMCs express Ase (blue). Progenitor cell streams (arrows) connect the proximal and distal IPC. (D-D″) Schematic of genetic mosaic screen. Asterisks indicate EMS-induced mutations. Unlike in wild type (wt; D′), in *3-78* ELF mosaics (D″), R1-R6 axons, labeled with *ro-τ-lacZ* (blue), mistargeted to the medulla (arrow), causing gaps (arrowheads) in the lamina plexus (brackets). R1-R8 axons are labeled with mAb24B10 (red). (E-H) Unlike in wild type (E,F), *3-78* ELF mosaics displayed large mutant Fas3-positive (red) NE cell clusters (arrowheads) at the third instar larval stage (G), that persisted into adulthood (H). (I) Meiotic recombination crosses of *3-78* separated three lethal mutations (arrowheads). 3-78*38 carried one mutation (asterisk), 3-78*56 contained two other mutations. (J,K) 3-78*38 ELF mosaics contained large (arrowheads) and small (arrows) ectopic Fas3-positive mutant NE cell clusters. (L) Genomic locus and protein structure of *Pcl* indicating the stop mutation (asterisk) in Pcl^3-78*38^. (M) In wild type, *h-lacZ* was expressed in the p-IPC, but not the OPC. The *mδ-lacZ* transgene within the ELF system labels R4 axons (arrow). (N) Ectopic Pcl^3-78*38^ NE cell clusters expressed *h-lacZ* (arrowheads). For additional information, genotypes and sample numbers, see Fig. S1 and Table S1. dc, distal cells (include C2, C3 and T2 neurons in adults); d-IPC, distal IPC; GPC, glial precursor cell areas; La, lamina; Lo, lobula; Lop, lobula plate; ln, lamina neurons (L1-L5 in adults); lopn, lobula plate neurons (T4 and T5 in adults); Me, medulla; mn, medulla neurons; os, optic stalk; p-IPC, proximal IPC; Re, retina; Tm and TmY, transmedullary neurons. Scale bars: 50 µm.

In the *Drosophila* visual system, photoreceptors (R-cells R1-R8) within the compound eye extend axons into the optic lobe consisting of four ganglia: the lamina, medulla, lobula plate and lobula (Fig. 1A). R-cell axons and highly diverse sets of target neuron subtypes are interconnected in a complex retinotopic map dedicated to processing visual information (Hadjieconomou et al., 2011). In contrast to other fly brain areas, these higher-order neurons are generated by two persisting postembryonic neuroepithelia, called the outer and inner proliferation centers (OPC and IPC; Fig. 1B,C) (Apitz and Salecker, 2014; Hofbauer and Campos-Ortega, 1990; White and Kankel, 1978). The OPC primarily gives rise to neurons associated with the lamina and medulla, and the IPC with the lobula plate and lobula (Hofbauer and Campos-Ortega, 1990). Both neuroepithelia are derived from the optic lobe placode, which arises during embryogenesis by invagination from the ectoderm and subsequently attaches to the lateral brain surface (Green et al., 1993). Already by the first instar larval stage, optic placode cells are partitioned into the two primordia (Apitz and Salecker, 2015). These initially expand by symmetric divisions into the two horseshoe-shaped OPC and IPC neuroepithelia (Egger et al., 2007; Nassif et al., 2003). During the late second instar larval stage, neurogenesis is initiated in the OPC, followed by the IPC (Hofbauer and Campos-Ortega, 1990). Recent studies uncovered that these neuroepithelia employ at least three distinct neurogenesis modes. First, NE cells at the lateral OPC edge give rise to lamina precursor cells (LPCs), which divide once to produce lamina neurons. Two R-cell axon-derived anterograde signals, Hedgehog (Hh) and the epidermal growth factor (EGF) homolog Spitz, promote the final division of LPCs and the generation and differentiation of lamina neurons, respectively (Huang and Kunes, 1996; Huang et al., 1998). Lamina neurogenesis further depends on the activity of the orphan nuclear receptor Tailless (Tll) (Guillermin et al., 2015) and retinal determination gene network (RDGN) members within LPCs (Pineiro et al., 2014). Second, NE cells at the medial OPC edge gradually convert into NSCs, called neuroblasts (Nbs), in a proneural wave, timely progression of which is controlled by several signaling pathways (Egger et al., 2010; Reddy et al., 2010; Yasugi et al., 2008, 2010). Nbs follow the common type I proliferation pattern by dividing asymmetrically to self-renew and produce ganglion mother cells (GMCs), which in a final division give rise to medulla neurons (Brand and Livesey, 2011; Egger et al., 2007). A series of temporal transcription factors – Homothorax (Hth), Eyeless (Ey), Sloppy paired (Slp), Dichaete (D) and Tll – controls subtype diversification of medulla Nb progeny (Li et al., 2013; Suzuki et al., 2013). Third, in a subdomain of the IPC, the proximal IPC (p-IPC), NE cells convert into progenitors by a mechanism that shares characteristics with epithelial-mesenchymal transition (EMT) (Apitz and Salecker, 2015). These progenitors migrate to a secondary proliferation zone, the distal IPC (d-IPC), where they mature into Nbs. Cross-regulatory interactions between D and Tll mediate a switch in Nb competence to generate two neuron populations: distal cells and lobula plate neurons (Apitz and Salecker, 2015). How the OPC and IPC are specified as distinct neuroepithelia and how this correlates with the control of their characteristic neurogenesis modes is currently unexplored.

To gain insights into the underlying mechanisms, we conducted a forward genetic mosaic screen for mutants that affected OPC and IPC development. We isolated a new allele of the epigenetic regulator *Polycomblike* (*Pcl*) that caused the formation of conspicuous ectopic NE cell clusters within the lobula plate area. Detailed analysis of observed phenotypes revealed that large clusters originated from the OPC and adopted IPC characteristics. *Pcl* is required for maintaining OPC identity by preventing ectopic expression of the Hox gene *Abdominal-B* (*Abd-B*) and thus interference with area-specific determinants. Our search for these factors uncovered that the optic lobe NE default state is IPC-like, whereas the RDGN members Eyes absent (Eya), Sine oculis (So) and Hth (Kumar, 2010) confer OPC identity to NE cells and concomitantly mediate coordinated Nb generation by direct conversion.

## RESULTS

### A genetic screen for determinants regulating OPC and IPC development

To uncover the molecular pathways that control NE patterning in the OPC and IPC, we performed an ethyl methane sulfonate (EMS)-based forward genetic mosaic screen for chromosome 2R using the ELF system (Fig. 1D; for details see supplementary Materials and Methods). This approach relies on three transgenes, *ey^3.5^-Gal80*, *lama-Gal4* and *UAS-FLP*, to generate homozygous mutant somatic clones in the optic lobe, while leaving wild-type activity in the eye (Bazigou et al., 2007; Chotard et al., 2005). Optic lobes were initially screened for R-cell projection pattern defects as a sensitive readout for patterning errors. Subsequently, optic lobes of mosaic animals that exhibited phenotypes were labeled with an antibody against the cell adhesion molecule Fasciclin 3 (Fas3) to distinguish the IPC and its offspring from the OPC (Apitz and Salecker, 2015; Hayden et al., 2007; Tayler et al., 2004). One mutant, *3-78*, as well as a derived line, 3-78*38, in which second site lethal mutations were identified by deficiency mapping and removed by meiotic recombination, displayed two remarkable phenotypes in ELF mosaics. Large ectopic Fas3-positive NE cell clusters formed in the lobula plate area during postembryonic development and persisted into adulthood. Moreover, small Fas3-positive clusters accumulated in close vicinity of OPC and p-IPC NE cells (Fig. 1E-K; Fig. S1A-D).

Complementation assays and sequence analyses (Fig. 1L) identified the affected gene as a novel mutant allele of *Pcl* (Lonie et al., 1994), which we named Pcl^3-78*38^. Pcl and its vertebrate homolog PHF1 belong to the highly conserved Polycomb group (PcG) family of chromatin-modifying proteins. These form two functionally distinct Polycomb repressive complexes, PRC1 and PRC2 (Muller and Verrijzer, 2009). Pcl joins the PRC2 complex to mediate high levels of histone 3 lysine 27 trimethylation and, thus, effective target gene silencing (Nekrasov et al., 2007; Sarma et al., 2008). Pcl^3-78*38^ carries a G-to-A base pair substitution in the open reading frame, resulting in a premature stop codon at amino acid position 536 (Fig. 1L). This truncates the second of two plant homeodomain (PHD) fingers, abolishing the interaction of Pcl with PRC2 (O'Connell et al., 2001). Another allele, *Pcl^27T7a^* (Gaytan de Ayala Alonso et al., 2007), caused similar Fas3-positive cell clusters in larval and adult optic lobes (Fig. S1E,F), confirming *Pcl* as the responsible gene. Finally, expression of a reporter transgene for the IPC-specific NE cell marker *hairy* (*h-lacZ*; Southall et al., 2013) provided additional evidence that Pcl^3-78*38^ mutant clusters consisted of NE cells with IPC identity (Fig. 1M,N).

### PcG members are required for the acquisition of OPC identity

**Fig. 2.**
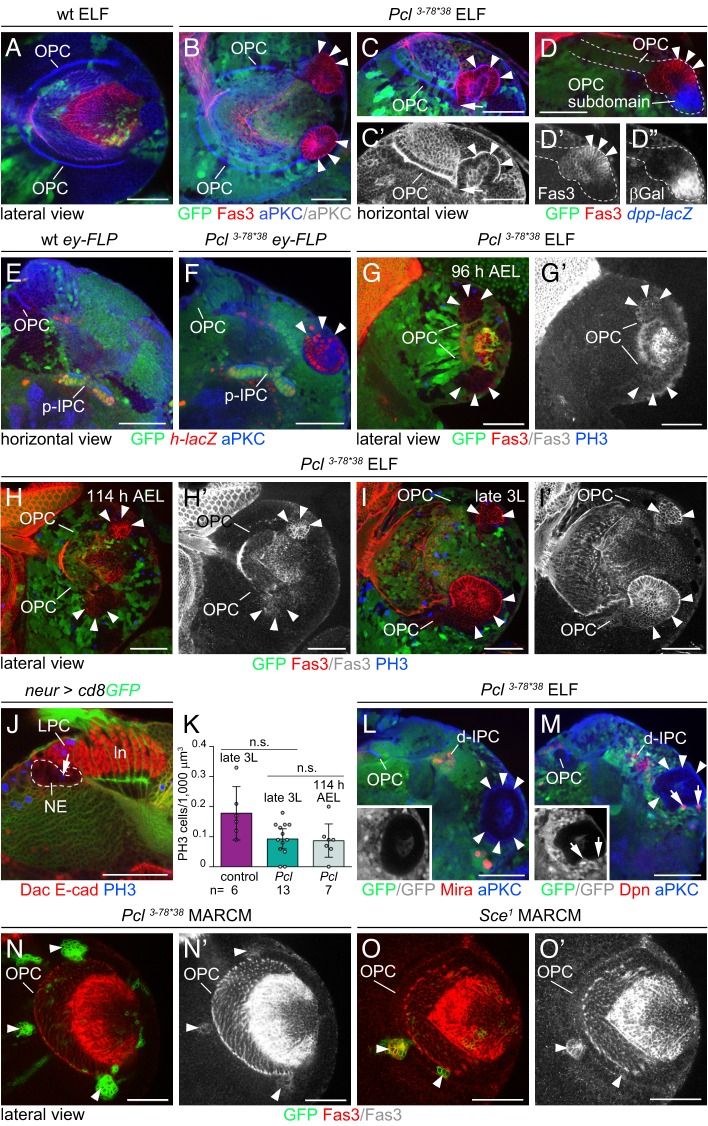
***Pcl* mutant NE cells change identity from OPC to p-IPC.** (A-C′) aPKC labeling (blue) of wild-type (A) and Pcl^3-78*38^ ELF mosaics (B-C′) showed that large Fas3-positive NE cell clusters (arrowheads) are continuous with the OPC (arrow in C,C′). (D-D″) Large cell clusters (red, arrowheads) arose adjacent to *dpp-lacZ* (blue)-labeled OPC subdomains. (E,F) In wild type (E), *ey-FLP* induces clone formation in the OPC, and not the IPC. Pcl^3-78*38^ clones induced in the OPC (F) form large NE cell clusters (arrowheads) expressing the p-IPC marker *h-lacZ* (red). (G-I′) OPC-derived ectopic NE cell clusters in Pcl^3-78*38^ ELF mosaics increase in size (arrowheads) and express increasing levels of Fas3 (red) at 96 h (G,G′) and 114 h (H,H′) AEL, and in late third instar (3L) larvae (I,I′). Few cells are labeled with PH3 (blue). (J) Dashed lines outline OPC neuroepithelium in *neur^P72^-Gal4 UAS-cd8GFP* (green) optic lobes labeled with Dac (red) and E-cad (red). Arrow indicates mitotic NE cell labeled with PH3 (blue). ln, lamina neurons. (K) The numbers of PH3-positive cells in control OPC neuroepithelia and Pcl^3-78*38^ clusters are similar in late third instar larvae (two-tailed unpaired Student's *t*-test, *P*=0.059) and at 114 h AEL (*P*=0.832). Graph shows data point distributions and means±95% confidence intervals. (L,M) In Pcl^3-78*38^ ELF mosaics, ectopic NE cell clusters (arrowheads; insets) did not give rise to Nbs labeled with Mira (red; L) and Dpn (red; M). Nbs in the vicinity (arrows, M, inset) are GFP positive and therefore heterozygous. (N-O′) Similar to Pcl^3-78*38^ MARCM clones in the OPC (N,N′), *Sce^1^* mutant NE cells ectopically expressed Fas3 (arrowheads) (O,O′). See also Fig. S1. For genotypes and sample numbers, see Table S1. Scale bars: 50 µm.

The expression of Fas3 and *h-lacZ* within large ectopic cell clusters in the lobula plate area suggested that these originated from the p-IPC. However, two observations support the notion that these clusters are derived from the OPC. First, 3D analysis of samples, stained with the cell polarity marker atypical protein kinase C (aPKC) to facilitate tracing of epithelial membranes, revealed that ELF system-induced clusters were continuous with the OPC neuroepithelium (Fig. 2A-C). Ectopic clusters formed immediately adjacent to the dorsal and ventral Decapentaplegic (Dpp)-expressing OPC subdomains (Fig. 2D; Kaphingst and Kunes, 1994). Second, to induce clone formation solely in the OPC, but not the IPC, we utilized *eyeless (ey)-FLP* (Newsome et al., 2000) as recombinase source (Fig. 2E). These Pcl^3-78*38^ clones formed large *h-lacZ*-positive NE cell clusters (Fig. 2F) at similar positions as those generated by the ELF system.

To test whether large clusters arose because of increased proliferation, optic lobes were labeled with the mitotic marker phosphoHistone 3 (PH3). Mutant clusters were discernible from the early third instar larval stage onwards (Fig. 2G-I′). Comparing Pcl^3-78*38^ mutant ectopic clusters with wild-type OPC NE cells in wandering third instar larvae, we observed a slight nonsignificant decrease in mitotic activity. Moreover, the average number of mitotic cells in ectopic clusters in early and wandering third instar larvae remained constant (Fig. 2J,K; Fig. S1G; for details, see supplementary Materials and Methods). Absence of staining with the Nb-specific markers Miranda (Mira) and Deadpan (Dpn) around Pcl^3-78*38^ mutant clusters indicated that these did not generate Nbs (Fig. 2L,M). Finally, using mosaic analysis with a repressible cell marker (MARCM; Lee and Luo, 1999) we found that, similar to Pcl^3-78*38^, OPC NE cells mutant for the PRC1 component *Sex combs extra* (*Sce*) ectopically expressed Fas3 (Fig. 2N,O). This observation indicates that *Pcl* functions within the context of PRC1 and PRC2. Thus, impaired *Pcl* function leads to the formation of large clusters not by affecting proliferation, but by altering the identity of OPC NE cells, including their ability to generate Nbs.

### PcG members repress Abdominal-B in the OPC

**Fig. 3.**
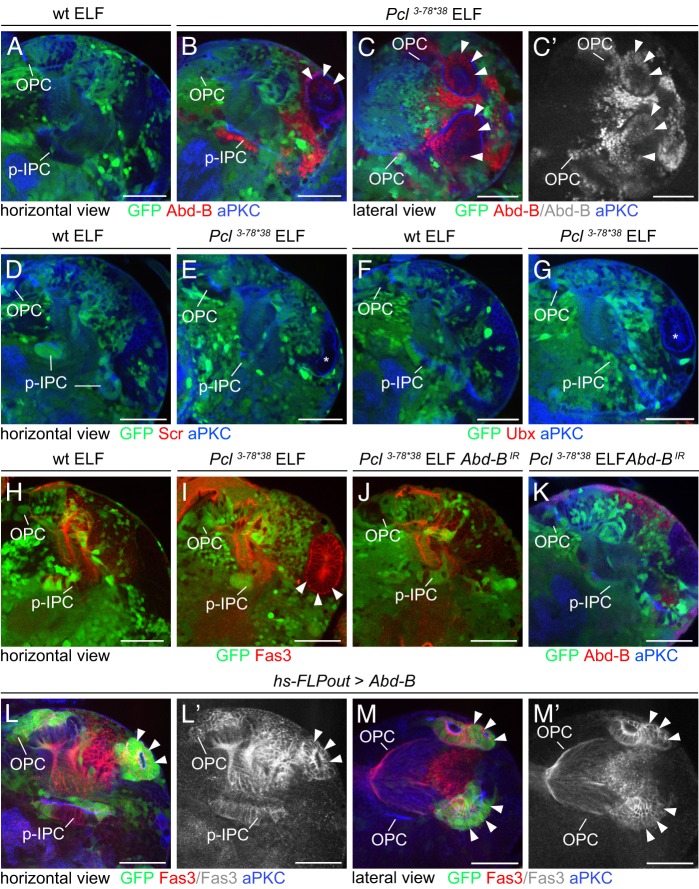
**Ectopic Abd-B mediates the identity change from OPC to p-IPC.** (A-G) Wild-type optic lobes do not express Abd-B (red, A), Scr (red, D) or Ubx (red, F). In Pcl^3-78*38^ ELF mosaics, Abd-B (red, B,C), but not Scr (E, red) and Ubx (G, red), were ectopically expressed in clones, including the large clusters (arrowheads). Asterisks indicate absence of Scr and Ubx expression in clusters. (H-K) Compared with wild-type ELF mosaics (H), formation of large ectopic Fas3-positive NE cell clusters in Pcl^3-78*38^ ELF mosaics (I, arrowheads) was suppressed by expression of *Abd-B^IR^* with *lama-Gal4* contained in the ELF system (J). Ectopic Abd-B was absent from NE cells in the OPC and p-IPC, and weakly expressed in neuronal progeny (K). (L-M′) *hs-FLPout* clones (green) expressing ectopic Abd-B formed large Fas3-positive NE cell clusters derived from the OPC (red, arrowheads). See also Fig. S2. For genotypes and sample numbers, see Table S1. Scale bars: 50 µm.

One key function of PcG proteins is to silence Hox genes (Lanzuolo and Orlando, 2012). These encode evolutionarily conserved homeodomain-containing transcription factors essential for patterning tissues along the anterior-posterior body axis (Peter and Davidson, 2011). Hox genes have to be tightly regulated in the areas where they are not normally active, because ectopic posterior homeotic genes can repress their anterior counterparts or interfere with the activity of signaling pathways and endogenous determinants (Gehring et al., 2009). Examining Abdominal-B (Abd-B), Sex combs reduced (Scr) and Ultrabithorax (Ubx) we observed that, unlike in wing imaginal discs (Fig. S2A-H), only Abd-B was ectopically expressed in Pcl^3-78*38^ mutant clones in the optic lobe (Fig. 3A-G). Knockdown of *Abd-B* in Pcl^3-78*38^ mutant clones impaired the formation of large Fas3-positive cell clusters (Fig. 3H-K). Overexpression of Abd-B using the FLPout approach in conjunction with an *hs-FLP* transgene (Ito et al., 1997) was sufficient to induce their formation (Fig. 3L,M). Together, this indicates that in *Pcl* clones, ectopic Abd-B mediates the change of NE cells from an OPC fate to an IPC-like fate.

### Ectopic Abd-B interferes with Decapentaplegic-dependent EMT and progenitor differentiation in the IPC

**Fig. 4.**
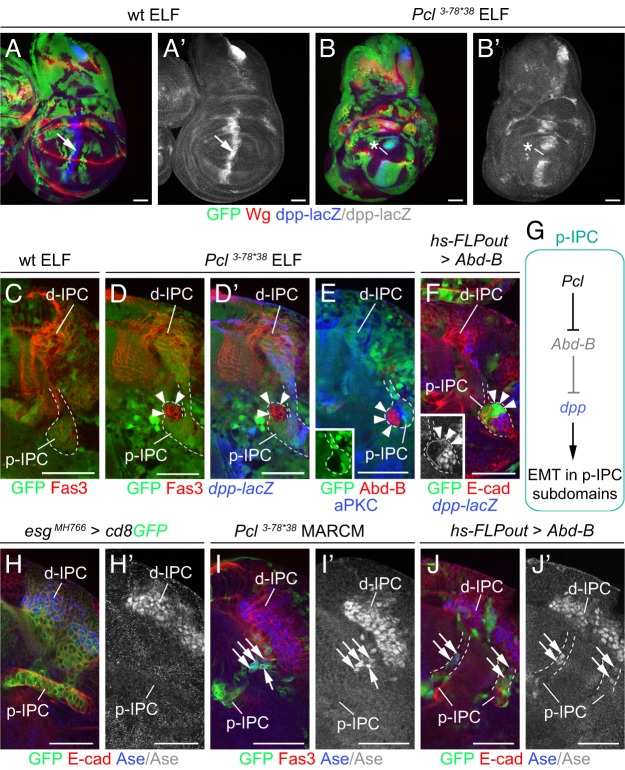
**Ectopic Abd-B affects Dpp-dependent EMT and progenitor differentiation in the IPC.** (A-B′) Unlike in controls (A,A′), *dpp-lacZ* expression (blue) at the anterior-posterior boundary (arrow) was repressed in Pcl^3-78*38^ ELF mosaic wing imaginal discs (B,B′, asterisk). (C-E) Unlike in controls (C), Pcl^3-78*38^ ELF mosaics (D) showed small Fas3-positive (red) clusters (arrowheads) close to the p-IPC (dashed line). These were *dpp-lacZ* negative (blue, D′) and Abd-B positive (red, E, inset). (F) *hs-FLPout* clones (green) expressing ectopic Abd-B formed small clusters (arrowheads) adjacent to p-IPC (dashed line) subdomains and downregulated *dpp-lacZ* (blue; inset, white; arrowheads). (G) Model of *Pcl* function in p-IPC subdomains. (H-J′) Progenitor cell streams between the p-IPC and d-IPC in *esg^MH766^-Gal4, UAS-cd8GFP* (green) animals did not express Ase (blue) (H,H′). Pcl^3-78*38^ mutant progenitors (green) generated by MARCM ectopically expressed Ase (blue, arrows) (I,I′). Ectopic expression of Abd-B using the *hs-FLPout* approach had the same effect (J,J′, arrows). For genotypes and sample numbers, see Table S1. Scale bars: 50 µm.

Loss of *Pcl* causes the formation of large ectopic OPC-derived clusters, as well as small clusters adjacent to the p-IPC (Fig. 1K). We previously had observed a similar phenotype upon removal of the BMP type I receptor *thickveins* (*tkv*), consistent with a requirement of the TGFβ family member Dpp for EMT in IPC subdomains (Apitz and Salecker, 2015). We therefore tested whether loss of *Pcl* or ectopic Abd-B could interfere with Dpp signaling. Consistent with known regulatory interactions between ectopic Hox gene expression and Dpp signaling in wing imaginal discs (Crickmore and Mann, 2006), Pcl^3-78*38^ clones failed to express *dpp-lacZ* at the anterior-posterior boundary (Fig. 4A,B). Similarly, small Pcl^3-78*38^ clusters adjacent to the p-IPC did not express *dpp-lacZ* (Fig. 4C,D), but upregulated Abd-B (Fig. 4E). Consistent with this, Abd-B overexpression was sufficient to repress *dpp-lacZ* in p-IPC subdomains (Fig. 4F). Thus, loss of *Pcl* and ectopic Abd-B interfere with Dpp-mediated EMT in a subset of p-IPC cells (Fig. 4G).

Furthermore, we had observed in MARCM experiments that migratory progenitors in the IPC that lacked *Pcl* prematurely differentiated into Asense (Ase)-expressing Nbs within cell streams (Fig. 4H,I; Apitz and Salecker, 2015). Again, ectopic Abd-B expression was sufficient to induce a similar phenotype (Fig. 4J). Hence, migratory progenitors within the IPC are prevented from maturing into Nbs by a mechanism that is sensitive to loss of *Pcl* and ectopic *Abd-B*. Defects observed following *Pcl* loss are primarily caused by ectopic *Abd-B* activity. Genetic manipulations of *Pcl* and *Abd-B* can therefore serve as powerful genetic tools to uncover the genes that distinguish the OPC from the IPC.

### RDGN members are specifically expressed in the OPC

**Fig. 5.**
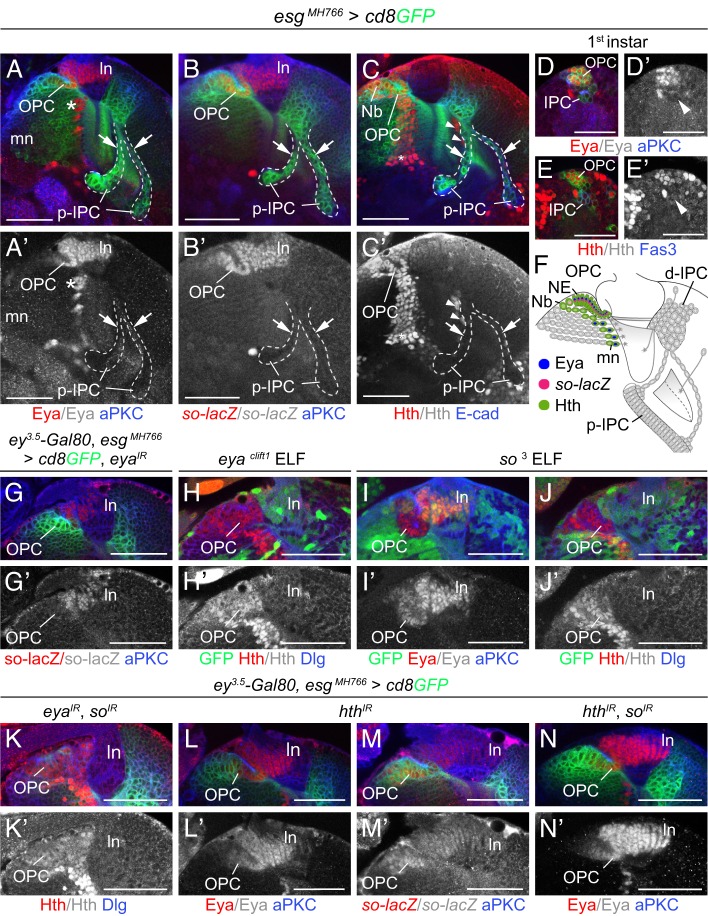
**RDGN member expression distinguishes OPC and IPC.** (A-E′) Optic lobes were labeled with *esg^MH766^-Gal4*, *UAS-cd8GFP* (green) and aPKC, E-cad or Fas3 (blue). At the third instar larval stage, Eya (red; A,A′), *so-lacZ* (red; B,B′) and Hth (red; C,C′) were detected in OPC but not IPC NE cells or cell streams (outlined; arrows indicate cell streams). Eya (A) and *so-lacZ* (B) were expressed in lamina neurons (ln), Eya in a subset of medulla neurons (mn, asterisks), and Hth (C) in some OPC Nbs, medulla neuron subtypes (asterisks) and glia (arrowheads). At the first instar larval stage, Eya (red; D,D′) and Hth (red; E,E′) were expressed in the OPC, and not the IPC (arrowheads; D′,E′). (F) Schematic of OPC-specific RDGN member expression. mn, medulla neurons. (G-N′) Cross-regulatory interactions of *eya*, *so* and *hth* in the OPC. Knockdown of *eya* resulted in the absence of *so-lacZ* expression (red; G,G′) in OPC NE cells. Hth (red; H,H′) was not affected in *eya^clift1^* ELF mosaics. Eya (red; I,I′) or Hth (red; J,J′) expression were not altered in *so^3^* mutant OPC NE cells. No mutant lamina neurons (ln) formed in *eya^clift1^* and *so^3^* ELF mosaics (H-J). Hth (red; K,K′) was not affected by simultaneous knockdown of *eya* and *so*. Eya (red; L,L) and *so-lacZ* (red; M,M′) were not impaired by *hth* knockdown. Simultaneous knockdown of *hth* and *so* did not affect Eya (red; N,N′) expression. See also Fig. S3. For genotypes and sample numbers, see Table S1. Scale bars: 50 µm (A-C′,G-N′), 25 µm (D-E′).

In eye-antennal disc epithelia, the RDGN bestows retinal identity (Kumar, 2010). Some members are also expressed and required in the optic lobe (Cheyette et al., 1994; Daniel et al., 1999; Pineiro et al., 2014; Southall et al., 2013). However, their expression had not been compared between the OPC and IPC, and their function in the medial OPC is not well understood. Using antisera or genetic markers, we detected the transcription factor and protein phosphatase Eya (Bonini et al., 1993), the SIX protein So (Cheyette et al., 1994) and the Meis/Prep homolog Hth (Rieckhof et al., 1997) in NE cells of the OPC, but not the IPC (Fig. 5A-C,F). Eya and Hth were specific to the OPC from the first instar larval stage onwards (Fig. 5D,E). At this stage, *so-lacZ* was expressed in the OPC and IPC (Fig. S3D), consistent with the previously described *so* requirement in embryonic optic placode formation (Cheyette et al., 1994) and perdurance of β-galactosidase expression. By contrast, the PAX family members Ey (Quiring et al., 1994) and Twin-of-Eyeless (Toy) (Czerny et al., 1999), as well as the transcriptional regulator Dachshund (Dac) (Mardon et al., 1994) and a reporter transgene of the zinc finger transcription factor Teashirt (Tsh) (Bessa et al., 2002) were not expressed in either neuroepithelium, but in specific Nbs and/or postmitotic neuronal progeny (Fig. S3A-B). Furthermore, the Six protein Optix which defines regional compartments in the OPC (Gold and Brand, 2014), was not detected in the IPC (Figs S3C and S4A).

Cross-regulatory interactions are a hallmark of RDGN function in the developing eye epithelium (Kumar, 2010). Therefore, we assessed RDGN member expression in OPC NE cells in *eya^clift1^* and *so^3^* clones, as well as *hth*, *eya* and *so* single or double knockdown experiments. Knockdown was achieved using validated UAS-RNA interference (RNAi) transgenes in combination with *esg^MH766^-Gal4* and *ey^3.5^-Gal80* transgenes (Fig. S3E-M′). These experiments revealed that in the OPC NE domain dedicated to generating medulla neurons, *so* expression depends on *eya* (Fig. 5G), but *eya* and *so* are not required for *hth* expression and vice versa (Fig. 5H-N).

### RDGN members contribute to medial OPC development

**Fig. 6.**
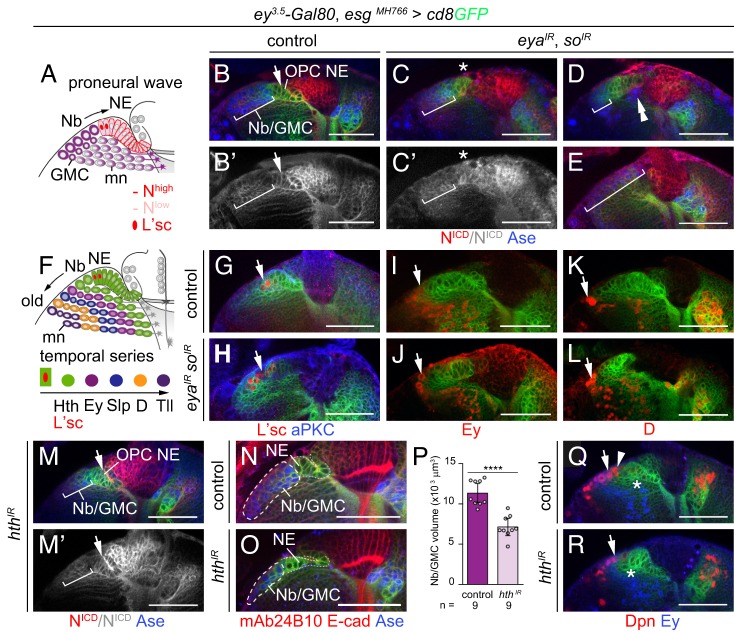
**RDGN members are required for controlled OPC Nb formation.** (A) Schematic of proneural wave progression and L'sc and N expression in the OPC. (B-E) In controls (B,B′), the intracellular domain of N (red) was detected at high levels in OPC NE cells (arrow), and at low levels in Nbs/GMCs (brackets) labeled with Ase (blue). Following simultaneous knockdown of *eya* and *so*, the boundary between high and low N was less sharp (asterisk; C,C′). Nbs/GMCs were mispositioned below NE cells (double arrowheads; D), and areas occupied by Nbs/GMCs were expanded while NE cells were missing (E). (F) Schematic illustrating the sequential expression of Hth, Ey, Slp, D and Tll in OPC Nbs. (G,H) Whereas in controls (G), L'sc (red, arrow) was detected in two cells in the NE/Nb transition zone, an increased number of cells expressed L'sc upon *eya*/*so* knockdown (H). (I-L) In controls (I,K) and upon *eya*/*so* knockdown (J,L), Nbs expressed Ey and D (red, arrows). (M,M′) N appeared to be unaffected by *hth* knockdown. (N,O) Compared with controls (N), *hth* knockdown (O) reduced the area occupied by Nbs/GMCs in the OPC (dashed line) labeled with Ase (blue). R-cell axons were stained with mAb24B10 (red), cell membranes with E-cad (red). OPC NE cells are demarcated by dashed lines. (P) Nb/GMC volume measurements in controls and upon *hth* knockdown. Graph shows data point distributions and means±95% confidence intervals; the two-tailed unpaired Student's *t*-test *P* value is *P*=0.000019. (Q,R) As in controls (Q), Dpn-positive Nbs (red) expressed Ey (blue, arrows) following *hth* knockdown (R). The number of Ey-negative, Dpn-positive Nbs (arrowhead) and the area preceding Ey-positive progeny (asterisks) were reduced. See also Fig. S3. For genotypes and sample numbers, see Table S1. Scale bars: 50 µm.

Based on the observed expression pattern, we next examined the role of *eya*, *so* and *hth* in the OPC neuroepithelium. Previous studies had shown that Notch (N) signaling maintains the NE state of the OPC, and that downregulation, in part mediated by the proneural factor Lethal of scute [L'sc; L(1)sc], is required for the timely conversion of medial NE cells into medulla Nbs (Egger et al., 2010; Ngo et al., 2010; Orihara-Ono et al., 2011; Reddy et al., 2010; Wang et al., 2011; Yasugi et al., 2010) (Fig. 6A). We observed that knockdown of *eya* and *so* specifically in the optic lobe induced phenotypes consistent with previously reported defects linked to N signaling (Egger et al., 2010). Compared with wild type, N expression appeared reduced and diffuse in OPC NE cells (Fig. 6B,C). Labeling optic lobes with the Nb/GMC-specific marker Ase showed that clusters of Nbs/GMCs were mispositioned in the medulla cortex, or areas containing these cell types were expanded within gaps in the OPC neuroepithelium (Fig. 6D,E). In controls, L'sc was expressed in one or two NE cells that converted next into Nbs (Fig. 6A,F) (Yasugi et al., 2008), but knockdown of *eya* and *so* increased the number of cells expressing this factor (Fig. 6G,H). As newborn Nbs mature, they successively express a series of temporal transcription factors (Hth, Ey, Slp, D and Tll) that control medulla neuron subtype differentiation in a birth order-dependent manner (Li et al., 2013; Suzuki et al., 2013) (Fig. 6F). Despite knockdown of *eya* and *so*, Nbs were able to express these markers (Fig. 6I-L; data not shown).

By contrast, knockdown of *hth*, which is expressed in OPC NE cells and Nbs, did not affect N expression (Fig. 6M), but reduced the volume of Nbs and GMCs in the OPC by ∼37% (Fig. 6N-P). Consistently, Ey-positive Nbs and their progeny were located in closer proximity to OPC NE cells (Fig. 6Q,R). This suggests that *hth* is required in OPC NE cells to provide input into the number of generated Nbs that can progress through the temporal cascade. Hence, *eya*, *so* and *hth* contribute to the specification of the OPC and its distinct neurogenesis mode by direct conversion of NE cells to Nbs.

### Loss of *Pcl* and ectopic *Abd-B* affect OPC-specific expression of RDGN members

**Fig. 7.**
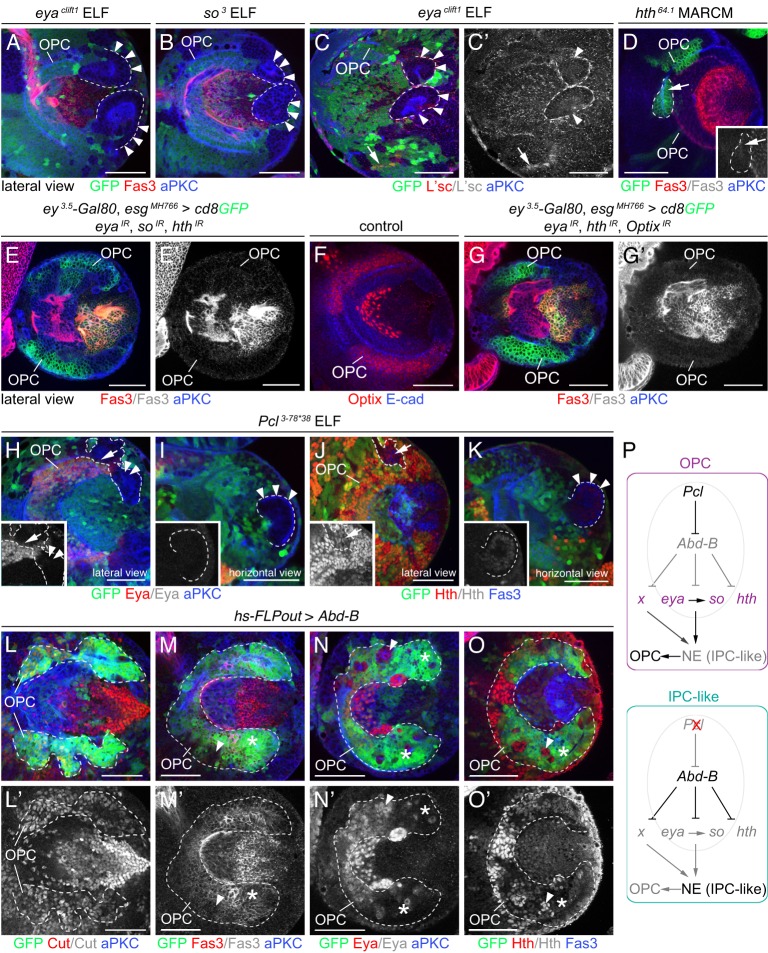
**Loss of *Pcl* and ectopic Abd-B downregulate the expression of OPC-specific RDGN members.** (A-C′) Mutant OPC NE cells in *eya^clift1^* (A) and *so^3^* (B) ELF mosaics formed large clusters (arrowheads; outlined), but did not express ectopic Fas3 (red). L'sc-expressing cells (red) were detected in the core OPC (arrow) but not in ectopic clusters (arrowheads; outlined) in *eya^clift1^* ELF mosaics (C,C′). (D) MARCM generated *hth^64.1^* mutant NE cells in the OPC (arrow; outlined) did not express Fas3 (red; white in inset). (E-G′) Simultaneous knockdown of *eya*, *so* and *hth* (E,E′) or *eya*, *hth* and *Optix* (G,G′) did not result in ectopic Fas3 expression (red) in OPC NE cells. In wild type, Optix (red) was expressed in dorso-ventral OPC subdomains (F). (H-K) In Pcl^3-78*38^ ELF mosaics, Eya (red; H,I; white in inset) and Hth (red; J,K; white in inset) were downregulated in the OPC crescent (arrows) and OPC-derived large ectopic NE cell clusters (arrowheads). (L-O′) *hs-FLPout* clones (green) expressing ectopic Abd-B in the OPC (outlined) did not affect Cut expression (red; L,L′), but led to upregulation (asterisks) of Fas3 (red; M,M′) compared with control cells (arrowheads). Eya (red; N,N′) and Hth (red; O,O′) were downregulated (asterisks), compared with control cells (arrowheads). (P) Model of *Pcl* function in the OPC. x, additional unidentified factor. See also Fig. S4. For genotypes and sample numbers, see Table S1. Scale bars: 50 µm.

Subsequently, we tested our hypothesis that loss of *Pcl* and ectopic *Abd-B* could cause the formation of large OPC-derived ectopic clusters with IPC-like identity by interfering with the expression of OPC-specific RDGN members. We observed that, in addition to defects within the OPC crescent, *eya^clift1^* and *so^3^* ELF mosaic clones formed large NE cell clusters, similar to those caused by loss of *Pcl* (Fig. 7A,B). Clusters failed to express L'sc (Fig. 7C), suggesting that the conversion into Nbs was blocked. However, they did not upregulate Fas3, indicating that the switch to an IPC identity was partial (Fig. 7A,B). Also, *hth*-deficient MARCM clones did not express Fas3 (Fig. 7D), excluding Hth as the sole determinant responsible for Fas3 suppression. To determine whether *so*, *eya* and *hth* act redundantly, we simultaneously knocked down their expression. Whereas optic lobe development was impaired, NE cells did not express ectopic Fas3 (Fig. 7E). Finally, we assessed a possible contribution of *Optix* (Fig. 7F; Fig. S4A,B). Although the simultaneous knockdown of *eya*, *hth* and *Optix* affected all four genes (because *so* depends on *eya*), it did not result in ectopic Fas3 expression (Fig. 7G). This indicates that RDGN members alone do not confer full OPC identity.

Next, we examined the effects of loss of *Pcl* and ectopic *Abd-B* on RDGN member expression. In Pcl^3-78*38^ ELF mosaics, Eya and Hth were decreased in OPC NE cells and in large ectopic cell clusters (Fig. 7H-K). Consistent with this, overexpression of Abd-B in the OPC using the *hs-FLPout* approach led to upregulation of Fas3 and downregulation of Eya and Hth (Fig. 7M-O). By contrast, the homeodomain-containing transcription factor Cut (Blochlinger et al., 1990), which is expressed in the OPC and IPC (Fig. S4C,D), remained unaffected (Fig. 7L). Hence, loss of *Pcl* and ectopic Abd-B specifically affected the expression of OPC-specific RDGN members. We propose that interference with these and additional determinants transform the OPC into an undifferentiated IPC-like neuroepithelium (Fig. 7P).

### *eya* and *so* are sufficient to induce OPC-like identity

**Fig. 8.**
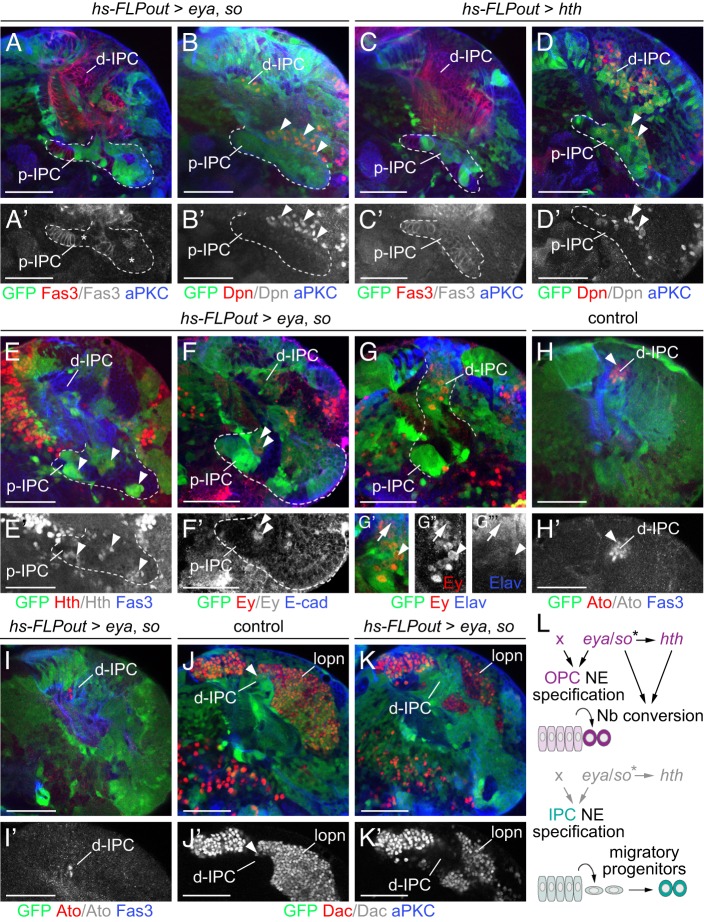
**Ectopic *eya/so* confer OPC-like identity to the p-IPC.** (A-B′) *hs-FLPout* clones (green) co-expressing ectopic *eya*/*so* downregulated (asterisks) Fas3 (red; A,A′) in the p-IPC (dashed line) and induced direct conversion into Dpn-positive Nbs (red; B,B′; arrowheads) from the p-IPC. (C-D′) Ectopic *hth* did not downregulate Fas3 (red; C,C′), but induced the formation of Dpn-positive Nbs (red; D,D′; arrowheads) in the p-IPC. (E-G′″) Ectopic *eya*/*so* induced Hth (red; E,E′; arrowheads) and Ey (red; F-G″) expression in the p-IPC. Ey was detected in Nbs (arrowheads) and in neuronal progeny expressing Elav (blue, arrow; G-G′″) within the d-IPC. (H-K′) In controls, the most distal Nbs (arrowheads) in the d-IPC expressed Ato (red; H,H′) and Dac (red; J,J′). Dac is maintained in lobula plate neurons (lopn). Clones expressing ectopic *eya/so* in the d-IPC did not express Ato (red; I,I′) or Dac (red; K,K′). (L) Model of *eya*, *so* and *hth* function in the OPC. Asterisk indicates sufficiency but not requirement of *eya/so* for *hth* expression. x, additional unidentified factor. See also Fig. S4. For genotypes and sample numbers, see Table S1. Scale bars: 50 µm.

Finally, to assess whether NE specification and neurogenesis are co-regulated, we monitored the effects of ectopically expressing *eya*, *so* and *hth* in the p-IPC neuroepithelium. In contrast to the OPC, the p-IPC gives rise to migratory progenitors, which subsequently mature into Nbs in the d-IPC. Whereas *eya* or *so* alone had only mild effects (Fig. S4E-H), co-expression of *eya* and *so* reduced Fas3 levels in p-IPC NE cells and, strikingly, induced the direct conversion of p-IPC NE cells into Dpn- and Mira-positive Nbs (Fig. 8A,B; Fig. S4I). Although ectopic Hth did not affect Fas3 levels (Fig. 8C), it triggered the conversion of p-IPC NE cells into Nbs (Fig. 8D; Fig. S4J). The proneural gene *l'sc*, the Notch target gene *E(spl)mγ* *HLH* and the EGF receptor target gene *pointed* (*pnt*) serve as proneural wave markers in the OPC (Yasugi et al., 2010). We observed that these genes were expressed in OPC and p-IPC neuroepithelia (Apitz and Salecker, 2015; Fig. S4K,L) and therefore could not be used as OPC-specific readouts. However, *eya* and *so* overexpression induced ectopic Hth (Fig. 8E), which also constitutes the first temporal series marker in the medulla (Li et al., 2013; Suzuki et al., 2013). Upon ectopic activation of Eya and So but not Hth, Nbs in the p-IPC, as well as Nbs and Elav-positive neuronal progeny in the d-IPC, expressed the second OPC-specific temporal marker Ey (Fig. 8F,G; Fig. S4M). Consistent with a progression through the OPC-specific temporal series, more Hth- than Ey-positive cells (Hth: 23.67±5.06 95% confidence interval; Ey: 4±1.4 95% confidence interval; *n*=12 optical sections from four optic lobes each) were found in the proximity of transformed OPC-like NE cells (unpaired, two-tailed Student's *t*-test, Welch corrected, *P*=1.42×10^−6^). Conversely, the d-IPC specific factors Atonal (Ato) and Dac failed to be expressed (Fig. 8H-K). Collectively, these findings show that *eya* and *so* play an instructive role in inducing OPC identity and in concert with *hth* promote Nb formation by direct transformation of NE cells, thus imposing an OPC-like mode of neurogenesis on the IPC (Fig. 8L).

## DISCUSSION

### Three roles of *Pcl* in optic lobe development

Chromatin-regulating proteins play pivotal context-dependent roles during neural development (Ronan et al., 2013). Despite the insights into the molecular function of Pcl within PRC2 (Nekrasov et al., 2007), our understanding of its *in vivo* roles in the CNS remained limited. Our findings uncovered central roles of *Pcl* in NE specification, EMT of migratory progenitors and their timely maturation into Nbs. Pcl does not regulate proliferation, and thus differs from some PcG members, such as the PRC1 components Posterior sex combs and Suppressor of zeste 2, loss of which causes tumor-like growth in imaginal discs (Classen et al., 2009) and the optic lobe (H.A. and I.S., unpublished). These roles are also distinct from those reported for other PcG genes in the central brain and VNC, as these regulate Nb survival and maintenance of neuronal identity in these contexts (Bello et al., 2007; Wang et al., 2006). *Pcl* mutant cells in the optic lobe specifically upregulated Abd-B, and loss of *Pcl* and overexpression of *Abd-B* caused qualitatively similar defects. This is consistent with the known posterior prevalence of Hox genes, whereby posterior genes are epistatic to anterior ones (Gehring et al., 2009). Thus, *Pcl* function in the optic lobe probably cannot be uncoupled from the fundamental developmental role of PcG members in silencing ectopic Hox expression. Although the mechanisms underlying Hox-mediated defects remain to be investigated, previous studies suggest that this may involve transcriptional repression (Vachon et al., 1992) or competitive protein-protein interactions (Gehring et al., 2009; Plaza et al., 2008, 2001).

### The IPC reflects a neuroepithelial default state in the optic lobe

The ability of *Pcl* loss to interfere with the activity of local transcriptional networks and signaling pathways revealed that the NE default state in the optic lobe is IPC-like, whereas OPC NE identity is derived. Several lines of evidence support this notion: First, IPC NE cells express the cell adhesion molecule Fas3 from the first instar larval stage onwards (Apitz and Salecker, 2015; Hayden et al., 2007; Tayler et al., 2004), whereas the RDGN members Eya and Hth are specific to the OPC with similar developmental timing. Second, *Pcl* mutant clusters originating from the OPC ectopically express Fas3 and *h-lacZ*. Interestingly, Fas3 was first identified as the antigen of a monoclonal antibody with high specificity to undifferentiated epithelial cells of imaginal discs (Brower et al., 1980). Third, the RDGN members Eya, So and Hth contribute to establishing the OPC as distinct neuroepithelium including the neurogenesis mode by direct NE-to-Nb conversion in the medulla. Eya and So together are sufficient to confer OPC-like identity on the p-IPC, which includes Fas3 downregulation. Fourth, ectopic Abd-B in *Pcl* mutant clones and in Abd-B gain-of-function experiments interfered with the expression of the OPC-specific genes *eya*, *so* and *hth*, and resulted in the formation of large NE cell clusters expressing IPC markers. Although our findings reveal effects for two neuroepithelia, they are consistent with the concept of neuronal homeosis and the relevance of recruiting determinants into new contexts to generate cellular diversity in the nervous system (Arlotta and Hobert, 2015).

Although the NE default state is IPC-like, full p-IPC differentiation and neurogenesis clearly depend on additional factors, such as Dpp signaling, which is required for EMT of migratory progenitors in p-IPC subdomains (Apitz and Salecker, 2015). Consistent with this, although large ectopic *Pcl* mutant NE cell clusters displayed a similar proliferation rate as wild-type OPC NE cells, they did not generate any neuronal progeny and persisted until adulthood. Two reasons may explain this lack of neurogenesis. Clusters are found in areas that may be spatially segregated from signals that promote neurogenesis in the IPC. Furthermore, ectopic Abd-B expression in the clusters may interfere with these signals, if they are present. This idea would be consistent with our observation that ectopic Abd-B affects Dpp signaling in the p-IPC, as well as maturation of progenitors into Nbs.

### RDGN members control OPC specification and direct NE-to-Nb conversion

Our genetic analyses revealed central roles for *eya* and *so* in medial OPC development. They are necessary for OPC specification and maintenance, because their knockdown interfered with coordinated NE-to-Nb conversion by directly or indirectly impairing N signaling in the OPC. Moreover, *eya* and *so* are sufficient for OPC specification, because ectopic expression in the IPC suppressed Fas3, altered the neurogenesis mode to that of the medial OPC, and triggered the generation of Nbs expressing two temporal series markers, Hth and Ey. However, *eya* and *so* are not required for Hth or Ey induction, and thus may not act directly upstream of the temporal series of transcription factors in medulla Nbs (Hasegawa et al., 2011; Li et al., 2013; Suzuki et al., 2013). Knockdown of *hth* reduced OPC Nb and GMC numbers without affecting N, and ectopic expression in the p-IPC triggered Nb formation by direct conversion. Therefore, in addition to neuron subtype specification (Hasegawa et al., 2011; Li et al., 2013; Suzuki et al., 2013), *hth* may independently influence the conversion and number of Nbs, potentially by also regulating OPC NE proliferation (Pineiro et al., 2014). *eya*, *so* and *hth* knockdown in different combinations did not lead to Fas3 upregulation in OPC NE cells, indicating that Eya, So and Hth function redundantly with additional factors. This does not include the Six family member Optix, as simultaneous knockdown of *Optix*, *hth*, *eya*, and consequently also *so* because of its dependence on *eya*, did not result in ectopic Fas3 expression in OPC NE cells.

Our findings provide additional evidence for the notion that in the *Drosophila* visual system, RDGN core components engage in versatile cross-regulatory interactions and subcircuits to control eye (Atkins et al., 2013; Silver and Rebay, 2005), lamina (Pineiro et al., 2014) and medulla development. For instance, in eye imaginal discs, anterior to the morphogenetic furrow, positive-feedback loops between *eya*, *so* and *ey* induce Dac and maintain Ey expression, whereas posterior to the furrow, So, upregulated by Eya, and Dac repress *ey* transcription (Atkins et al., 2013). Ey is not expressed in the lamina, but acts as a member of temporal transcription factors in medulla Nbs. Although *eya* and *so* can induce the ectopic formation of Ey-positive Nbs in the p-IPC, they are not required for Ey expression in medulla Nbs. In the lamina, *eya* and *so* cooperate with R-cell axon-derived Hh signaling to activate the core RDGN member Dac, which in turn represses *hth* (Chotard et al., 2005; Huang and Kunes, 1996; Pineiro et al., 2014). By contrast, in the medulla, *eya* and *so* do not interact with Dac, and are not essential for *hth* regulation, expression of which is maintained in NE cells and first-born medulla Nbs.

Eya and So play a central role in distinguishing the OPC from the IPC. Although cranial placodes and neural crest are vertebrate innovations (Northcutt, 2005), Eya and Six proteins could play an analogous role in vertebrates by delineating adjacent epithelial head territories that generate sensory placodes from those dedicated to neural crest and epidermis (Christophorou et al., 2009). Six and Eya have been discussed as a driving force for the formation of vertebrate placodes by the acquisition of a novel function in NE patterning and proliferation in addition to its ancient function in neuronal differentiation to generate a larger density of specialized neurons (Schlosser et al., 2014). Interestingly, recent studies uncovered the existence of neurogenic proto-placodal ectoderm expressing the Six1/2 and Eya homologs in the tunicate *Ciona intestinalis* (Abitua et al., 2015), suggesting a conserved pre-vertebrate role of these two genes in regional patterning of epithelia with neurogenic potential. These and our findings support the notion that the ancestral gene regulatory cassette of Eya and So may have been re-employed several times to impart specific cellular properties, including neuroepithelial specification, during invertebrate and vertebrate evolution.

## MATERIALS AND METHODS

### *Drosophila* stocks and husbandry

*Drosophila melanogaster* crosses were maintained in standard medium at 25°C except for RNAi experiments, for which progeny were shifted to 29°C at 24 h after egg laying (AEL). Expression and functional studies were conducted using combinations of the *Gal4/UAS*, the *FLP/FRT* system-based ELF (Bazigou et al., 2007; Chotard et al., 2005), MARCM (Lee and Luo, 1999), FLPout (Ito et al., 1997; Struhl and Basler, 1993) and RNAi approaches. Pcl^3-78*38^ was isolated in a forward genetic mosaic screen. Detailed descriptions of parental stocks and crosses, conditions for clone induction, the EMS screen and deficiency mapping, as well as full genotypes and numbers of samples shown in main and supplementary figures are provided in supplementary Materials and Methods and in Tables S1 and S2.

### Molecular biology

Genomic DNA extraction, PCR and sequence analysis were performed following standard protocols. To determine the EMS-induced mutation in 3-78*38, the following primers were used to amplify genomic DNA from FRT42D/FRT42D 3-78*38 flies: 5′-GGCGTACCGCTTTTGTTTTA-3′ [Pcl-1F] and 5′-GATTGATTTGTCCCGCAGTT-3′ [Pcl-1R]; 5′-TCAAGGCCAACAACATACGA-3′ [Pcl-2F] and 5′-GCTTCAGTGGCAACTCATCA-3′ [Pcl-2R].

### Immunolabeling and imaging

Brains were dissected in PBS, fixed for 1 h at room temperature in 2% paraformaldehyde in 0.1 M L-lysine (Sigma-Aldrich) containing 0.05 M sodium phosphate buffer (pH 7.4), and washed in PBS containing 0.5% Triton X-100 (Sigma-Aldrich) (for details of staining protocol, see Shimosako et al., 2014). Primary and secondary antibodies used in this study are described in supplementary Materials and Methods. Images were collected with Zeiss/Bio-Rad Radiance 2100 and Leica TCS SP5 II confocal laser scanning microscopes and processed using Adobe Photoshop.

### Quantification and statistics

Sample numbers for each experiment in this study are provided in Tables S1 and S2. Strategies to determine: (1) OPC volumes of wild-type and *UAS-hth^IR^*-expressing animals, (2) the numbers of PH3-positive cells in wild-type OPC NE cells or Fas3-positive Pcl^3-78*38^ mutant cell clusters, (3) the number of *Pcl*-deficient migratory progenitors that prematurely differentiate into Nbs, and (4) the numbers of Hth- and Ey-positive cells in *eya/so* gain-of-function experiments are described in detail in supplementary Materials and Methods. Calculations of 95% confidence interval error bars and unpaired two-tailed Student's *t*-test *P* values were performed using Microsoft Excel software [Confidence.T and T.Test (type 3, not assuming equal variance)]. Prism 6 GraphPad was used to perform Shapiro–Wilk and D'Agostino–Pearson omnibus normality tests and to present quantifications as scatter plots and bar graphs. *P*<0.05 was considered to be statistically significant; ****P<0.0001.
